# Supplementary material for: Comparative Metabolomic Analysis Reveals Distinct Flavonoid Biosynthesis Regulation for Leaf Color Development of Cymbidium sinense ‘Red Sun’
Source: Int J Mol Sci. 2020 Mar 9;21(5):1869. doi: 10.3390/ijms21051869 (PMC7084835; doi:10.3390/ijms21051869)

## Slide 1
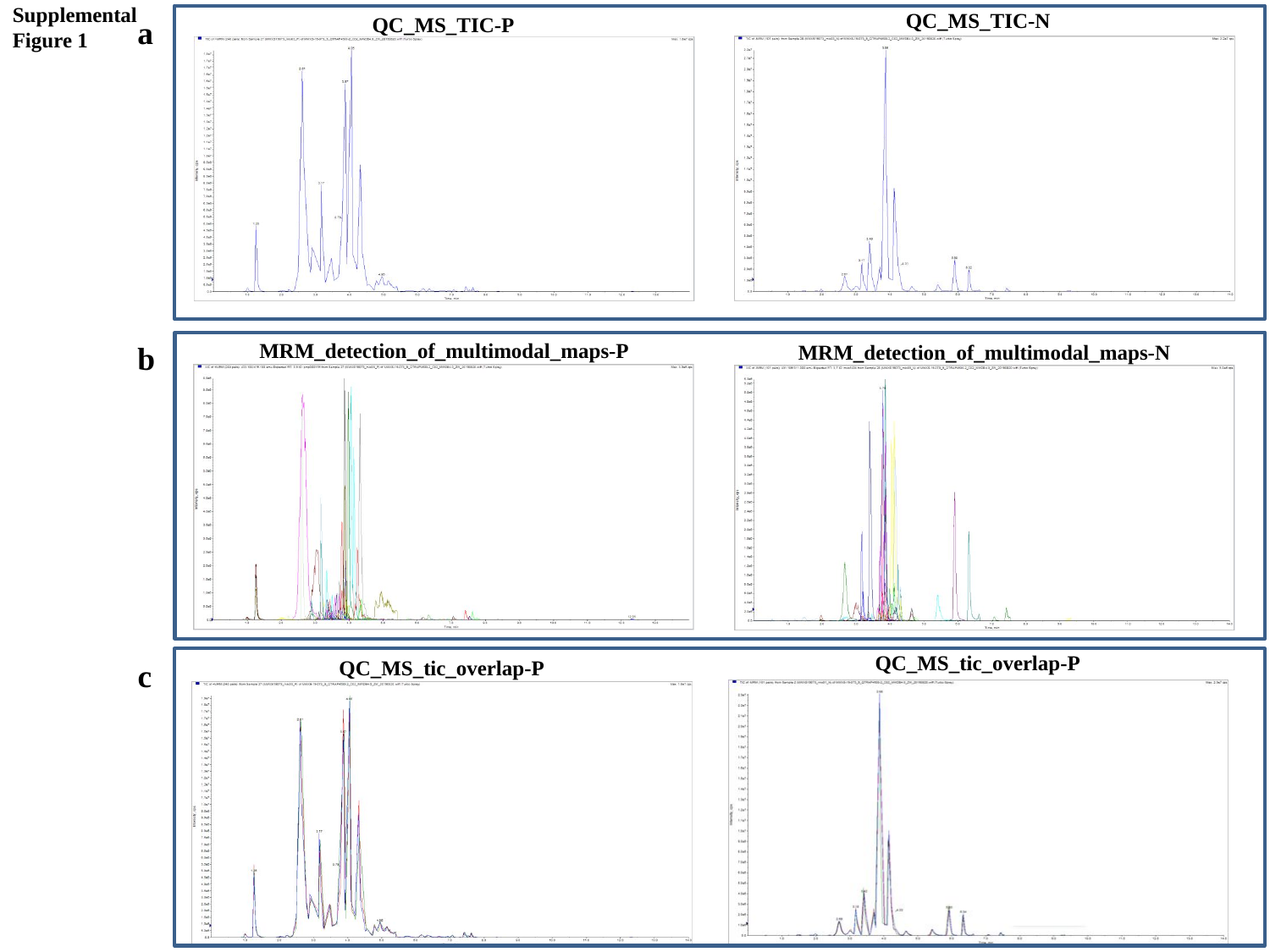

Supplemental Figure 1
QC_MS_TIC-N
QC_MS_TIC-P
a
MRM_detection_of_multimodal_maps-P
b
MRM_detection_of_multimodal_maps-N
QC_MS_tic_overlap-P
QC_MS_tic_overlap-P
c

## Slide 2
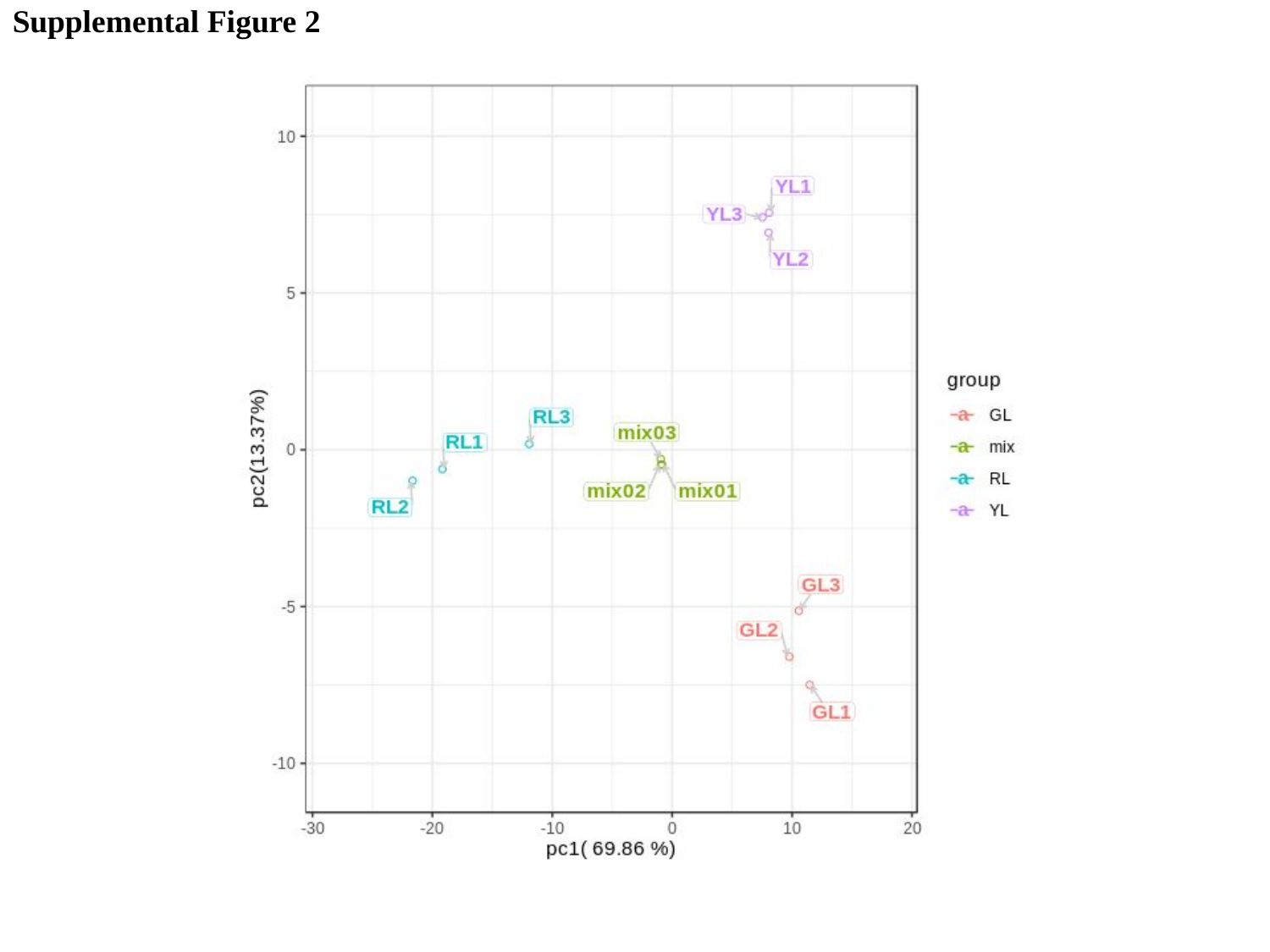

Supplemental Figure 2

## Slide 3
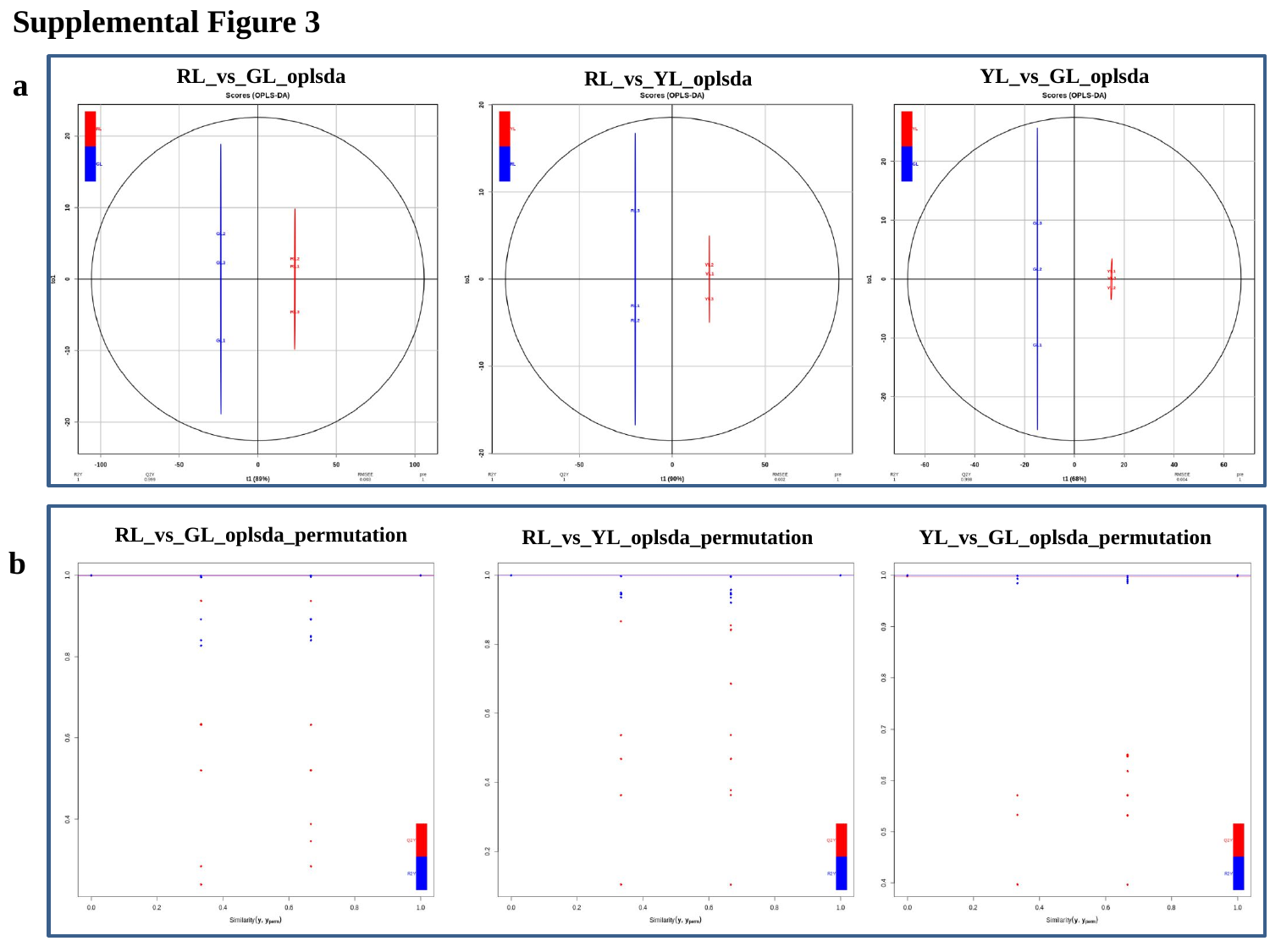

Supplemental Figure 3
RL_vs_GL_oplsda
YL_vs_GL_oplsda
a
RL_vs_YL_oplsda
RL_vs_GL_oplsda_permutation
YL_vs_GL_oplsda_permutation
RL_vs_YL_oplsda_permutation
b

## Slide 4
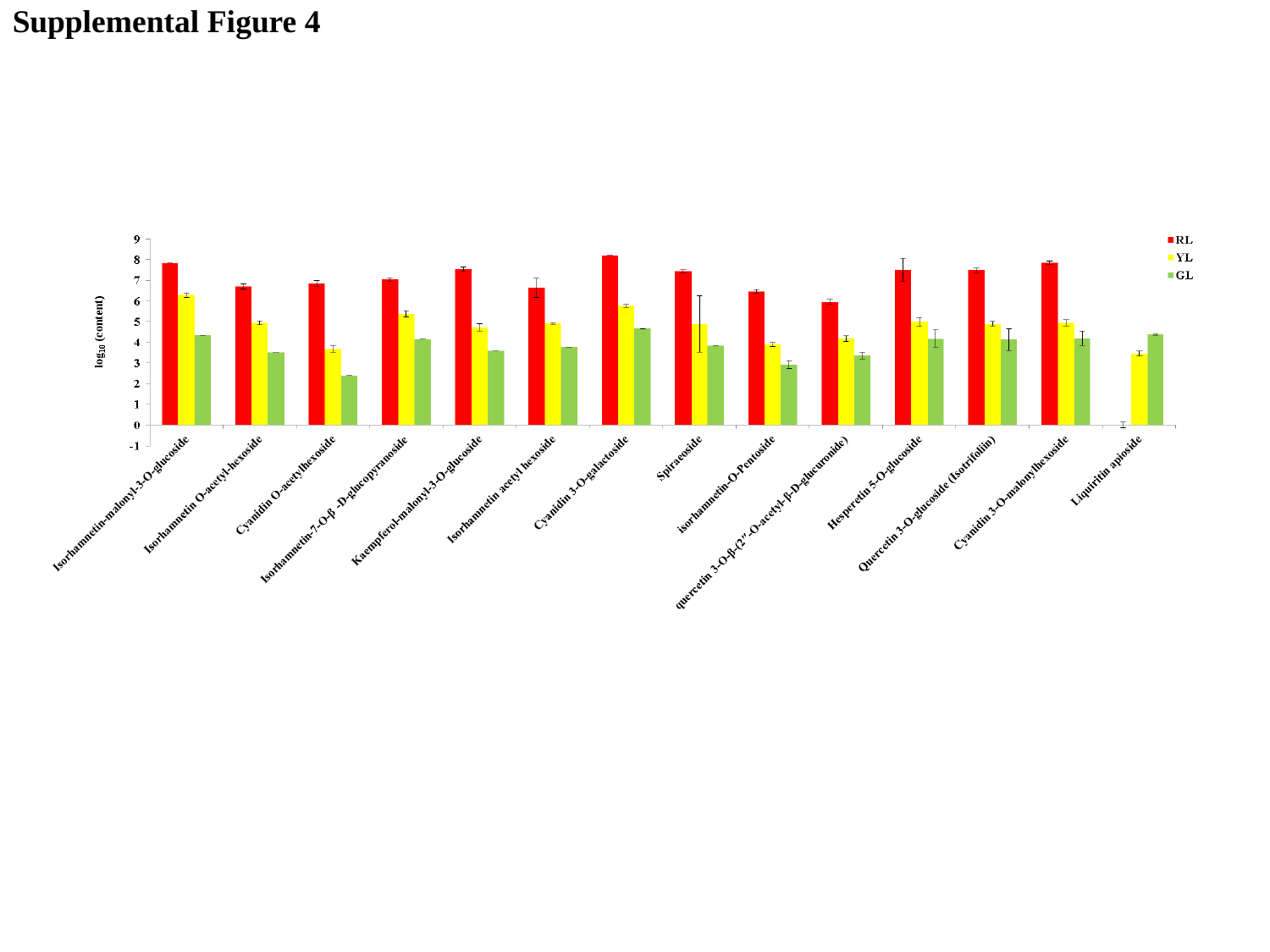

Supplemental Figure 4

## Slide 5
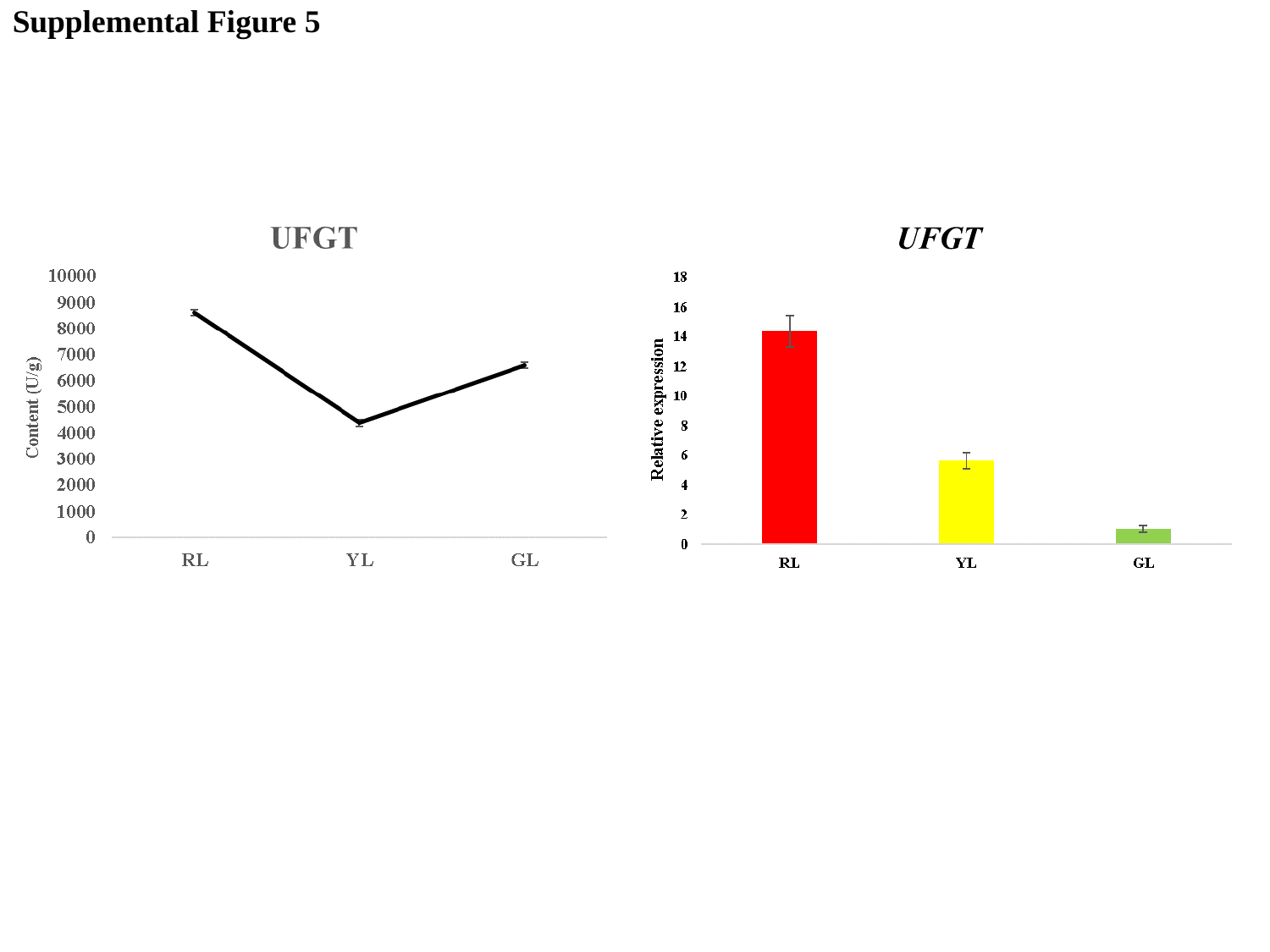

Supplemental Figure 5

## Slide 6
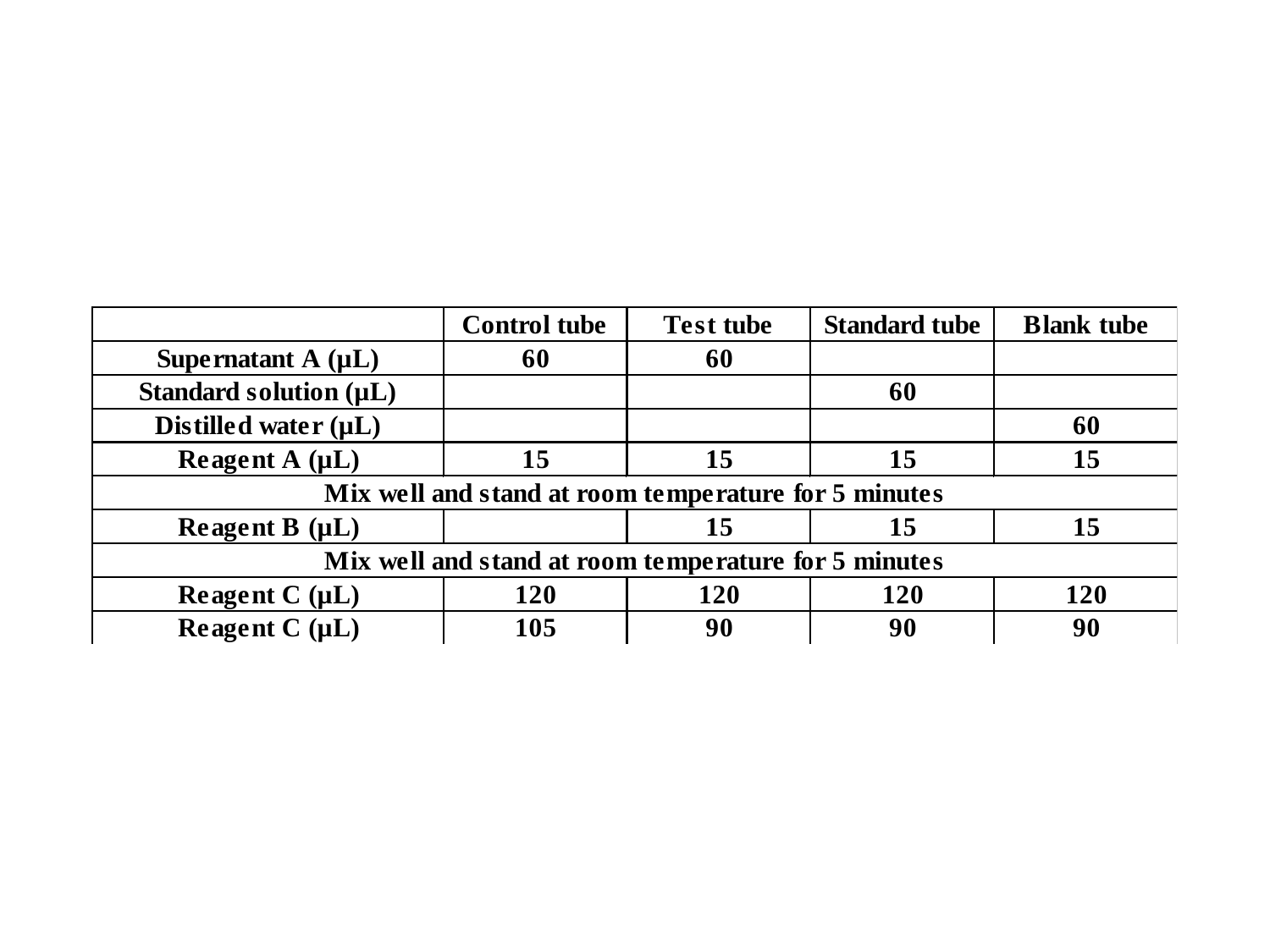

Supplement: Supplementary file 1 [file ijms-21-01869-s001.zip › Supplementary Files/Supplemental Figure.pptx]
