# Supplementary material for: Comparative Metabolomic Analysis Reveals Distinct Flavonoid Biosynthesis Regulation for Leaf Color Development of Cymbidium sinense ‘Red Sun’
Source: Int J Mol Sci. 2020 Mar 9;21(5):1869. doi: 10.3390/ijms21051869 (PMC7084835; doi:10.3390/ijms21051869)
Supplement: Supplementary file 1 [file ijms-21-01869-s001.zip › Supplementary Files/Supplemental Table 5.docx]

**Supplemental Table 2. Primers used for gene cloning and qPCR**

| **Genes** | **Forward primer (5'to 3')** | **Forward primer (5'to 3')** |
| --- | --- | --- |
| *CsPAL* | TGGCTTTTGAAAGGGGGTGG | GGCACTCAAGCAGGGAATCA |
| *CsCH4* | CACCGAGCCGGACATTCCA | GGTACTGCTCCGGGTTCTTC |
| *Cs4CL-1* | TGTTGATCGGGTGAAGGAGC | CACCACCTGCTTGGCTATGA |
| Mol018354 | ATGGACTCCTCCTCGGGCTCC | TCAGTAACTCCACAGAGAAAA |
| Mol010676 | ATGGGCGATCGCCGTCGTGCC | AGAATCTCTGGTATTTCGATTAG |
| Mol012054 | ATGGCCCCCAGGGAAAAGGGA | TCAGTTGAACTCAGCCGGAGG |
| Mol012056 | ATGATGGCAGTCGATACTCTT | TCAAAGGAGAGGCGGTGGAAGAT |
| Mol016418 | ATGGCCAACTCCGCTCCTCCATC | TTACTCGTCGAACTTCCATAAAT |
| Mol021163 | ATGGCCGACCCCACTCCGACT | CTACTCATCATCGAACTTCCA |
| Mol002504 | ATGGTGCCCAATCAGCAACCC | TCATCGTCGGGTATCATTCGG |
| Mol010095 | TCCCTACAAAACCCCGAAGA | AAGTGCCGAGCCAGATGC |
| Mol021020 | GCAGACGATGTGCCAATG | GCTCCAAATGTTCTTCCTGAT |
| Mol022332 | ATGAACAGACCCGACGAGC | GGAGACGGACGCCCTTATAG |
| Mol026160 | CTCAGACGAGGGCTCCTACA | CAGCGGACGCCTTTGTAGAT |
| Mol004997 | CGGTGACTCCAACTCCAAG | CTAATCGAAATACCAGAGATTCT |
| Mol019297 | GTTCAGAGGGTCAAAGGCGA | GGAGGAATAGGGGTTGCGAG |
| Mol002256 | GCGAGGAGAAGTCGGTTTCA | CCCAGCCATTTCCTCGTCTT |
| *CsACTIN* | CAATGAGCTTCGTGTTGCCC | GATACGAACCAGTTGTGCGG |
